# Development of a Safe and Effective mRNA Candidate Vaccine Against PEDV G2c Genotype Infection

**DOI:** 10.3390/v17091210

**Published:** 2025-09-04

**Authors:** Shixuan Zhu, Nan Cao, Huawei Zhang, Leqiang Sun

**Affiliations:** 1 State Key Laboratory of Agricultural Microbiology, Huazhong Agricultural University, Wuhan 430070, China; zsx123@webmail.hzau.edu.cn (S.Z.); caonan523523@webmail.hzau.edu.cn (N.C.); zhw@mail.hzau.edu.cn (H.Z.); 2 College of Veterinary Medicine, Huazhong Agricultural University, Wuhan 430070, China; 3 COFCO Joycome Foods Limited, Beijing 100000, China

**Keywords:** porcine epidemic diarrhea virus, mRNA vaccine, safety and efficacy

## Abstract

Porcine epidemic diarrhea virus (PEDV) is a highly contagious coronavirus that causes severe diarrhea, dehydration, and high mortality in piglets, leading to significant economic losses in the swine industry. The spike (S) protein of PEDV is the primary target for neutralizing antibodies and is critical for vaccine development. In this study, the pUC57-S01 and pUC57-S02 plasmids carrying the codon-optimized truncated S gene sequence were constructed. The mRNA S01 showed higher protein expression in vitro than mRNA S02, as confirmed by Western blotting. The safety and immunogenicity of mRNA S01 were evaluated in animal experiments. The results indicated that the mRNA S01 vaccine was safe for piglets and pregnant sows. Immunogenicity was assessed by a neutralization assay, which revealed that encapsulated mRNA S01 induced high levels of neutralizing antibody titers in pigs. Challenge protection efficiency tests showed that the mRNA S01 vaccine conferred immunity to newborn piglets, protecting them from a homologous PEDV strain challenge. This study provides a foundation for the clinical application of PEDV mRNA vaccines and offers a reference for the development of novel vaccines against PEDV.

## 1. Introduction

China is the world’s largest pig farming nation, with both the highest inventory and the highest slaughter volume globally. However, swine diseases have consistently been a major challenge hindering the healthy and sustainable development of the industry. Research shows that outbreak of severe porcine viral diarrhea diseases causes serious damage to the pig farming industry in China [1]. The causative factors of diarrhea are diverse, with viral factors accounting for the highest proportion. Virus-induced diarrhea is characterized by acute onset and high mortality rates. The main symptoms of viral diarrhea in pigs include watery diarrhea, vomiting, dehydration, and death [1,2]. The mortality rate of viral diarrhea can reach 100% in suckling piglets within 7 days of age. The primary pathogens responsible for porcine viral diarrhea include porcine epidemic diarrhea virus (PEDV), transmissible gastroenteritis virus (TGEV), porcine rotavirus (PRoV), porcine deltacoronavirus (PDCoV), and getah virus (GETV) [1,2,3,4,5]. Notably, PEDV causes the most severe damage to pig farms. PEDV belongs to the family Nidovirales, subfamily Coronavirinae, and genus Alphacoronavirus. Its virus particles have a diameter of 95 to 190 nm and an enveloped, single-stranded positive-sense RNA virus with a genome size of approximately 28 kb [6]. The genotypes of PEDV can be classified into G1 and G2 types, with G1 further divided into G1a and G1b subtypes, and G2 subdivided into G2a, G2b, and G2c subtypes [7,8]. Since 2010, significant changes have occurred in the epidemiological characteristics and incidence of PEDV, with the G2 variant strain becoming predominant [9].

The PEDV S protein, located on the outer viral envelope, is a homotrimeric type I glycoprotein. Numerous studies indicate that the S protein exhibits strong immunogenicity, capable of inducing high-titer neutralizing antibodies and cellular immune responses in hosts [10,11,12,13]. Thus, it is an ideal target for developing a novel vaccine against PEDV.

mRNA vaccines demonstrate notable advantages, including rapid intracellular expression, high efficacy and safety, and ease of large-scale production [14]. During the COVID-19 pandemic in 2020, mRNA vaccines played a crucial role, with the Moderna and BioNTech mRNA vaccines further validating safety and protective efficacy, leading to increased attention paid to mRNA vaccine technology [15,16]. Compared with other types of vaccines, the primary advantage of mRNA vaccines lies in their rapid development capability once the pathogen’s genetic sequence is obtained. mRNA vaccines efficiently induce both humoral and cellular immune responses whereas activating innate immune responses. They show promising application prospects in infectious disease prevention, cancer immunotherapy, and gene replacement therapy [17,18,19].

The limited protection offered by existing PEDV vaccines is due to their low protective efficacy [4,20,21]. The main strain of existing commercial vaccines is PEDV G2b, which cannot provide effective protection against the currently prevalent strains (PEDV G2a or PEDV G2c). In this research, the full-length PEDV S gene was selected as the target to develop a PEDV S mRNA vaccine and its protective efficacy was evaluated. Using cationic lipid nanoparticles (LNPs) to deliver PEDV S mRNA into pigs, the immune efficacy of the candidate PEDV S mRNA vaccine will be evaluated by neutralizing antibody titer, through experiments on pigs challenged with a virulent PEDV strain. This research aims to establish a foundation for the clinical applications of PEDV mRNA vaccines and provide novel strategies for developing next-generation PEDV vaccines.

## 2. Materials and Methods

### 2.1. Cells, Viruses, and Antibodies

Vero cells (ATCC; CCL-81), porcine kidney cells (PK-15, ATCC, CCL-33), and human embryonic kidney cells (293T, ATCC, CRL-3216) were grown in Dulbecco’s modified essential medium (DMEM; Invitrogen, Carlsbad, CA, USA) containing 10% fetal bovine serum (Gibco, New York, NY, USA) at 37 °C in a humidified 5% CO_2_ incubator. The PEDV NMG20-3 strain and NMG22-2 strain used in this study were isolated from diarrheal pigs in Inner Mongolia via Vero cells (Figure S1). The NMG20-3 strain belongs to G2c, whereas the NMG22-2 strain belongs to G2a. Monoclonal antibody (anti-S mAbs) was produced in our laboratory. Alexa Fluor 488 goat anti-mouse was obtained from Life Technologies, USA (Life Technologies Corporation, Carlsbad, CA, USA). Horseradish peroxidase (HRP)-conjugated goat anti-mouse IgG was obtained from Sigma-Aldrich Co (Sigma-Aldrich Corporation, Saint Louis, MO, USA).

### 2.2. Construction of Recombinant Plasmids

**Figure 1:**
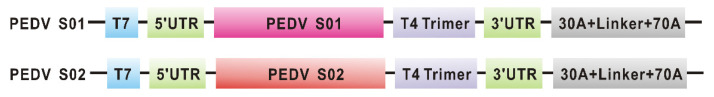
Schematic of the mRNA PEDV S01 and mRNA PEDV S02.

The S gene from the NMG20-3 strain (G2c) was synthesized as two sequences with codon optimization (Genscript Biotech Corporation, Nan Jing, China). These sequences carry their own signal peptide without a transmembrane domain, and a Trimer-Tag (T4 foldon) was added to the C-terminus [22]. The codon-optimized PEDV S01 and S02 genes were cloned into the plasmid pUC57-UTR with BamHI and EcoRI restriction site (in our laboratory), which contains 5′ and 3′ untranslated regions (human β-globin UTRs) and a poly A tail (110 bp) (Figure 1). The constructed plasmids were named pUC57-S01 and pUC57-S02, respectively.

### 2.3. mRNA Vaccine Preparation

The pUC57-S01 and pUC57-S02 plasmids were individually digested with BsPQI. The mRNA was synthesized in vitro using T7 polymerase-mediated transcription from the linearized plasmid DNA template with a T7 in vitro transcription kit (Cellscript Inc., Madison, WI, USA). In preparing the reaction system, UTP was replaced with N1-methylpseudoUTP. The final mRNA incorporated a cap 1 structure modification to enhance mRNA translation efficiency. The mRNA solution was dissolved in 50 mmol/L citrate buffer (pH 4.0).

LNP formulations were prepared by ethanol drop nanoprecipitation in a microfluidic mixer. SM-102, cholesterol, DSPC, and PEG2000-DMG were dissolved in ethanol at a molar ratio of 50:38.5:10:1.5. The lipid solution was mixed with the mRNA solution at a volume ratio of 1:3 using the microfluidic mixing method to form LNPs. The assembled LNPs were dialyzed in phosphate buffer (pH 7.4) to remove ethanol and exchange the buffer. The concentrated LNPs were filtered through a sterile filter (0.22 µm) and stored in Tris containing 2% sucrose. The product was characterized by particle size and distribution using a particle size analyzer (Malvern Panalytical, Nottingham, UK) and RNA concentration using HPLC (Thermo Fisher Scientific, Waltham, MA, USA).

### 2.4. Indirect Immunofluorescence Assay

293T cells were seeded into 24-well plates and transfected with 5 μg of PEDV S01 or PEDV S02 mRNA to form cell monolayers using Lipofectamine 2000 Transfection Reagent (Thermo Fisher Scientific, USA). After 36 h post-transfection, the cells were fixed in 4% paraformaldehyde for 20 min, permeabilized with 0.1% Triton X-100 at room temperature for 10 min, and then blocked with 2% bovine serum albumin. The cells were subsequently incubated with PEDV S-specific monoclonal antibodies (1:200 dilution), and the fixed cells were incubated with Alexa Fluor 488 goat anti-mouse (1:500 dilution). Fluorescence was observed under an Olympus IX73 fluorescent microscope (Olympus Corporation, Tokyo, Japan).

### 2.5. Western Blotting

The 293T cells or PK-15 cells were seeded into 6-well plates and transfected with 10 μg of PEDV S01 or PEDV S02 mRNA using Lipofectamine 2000 Transfection Reagent (Thermo Fisher Scientific, USA). After 48 h post-transfection, the protein concentration of whole-cell lysates was determined using the Micro BCA™ Protein Assay Kit (Thermo Scientific, USA). The protein samples were separated by sodium dodecyl sulfate–polyacrylamide gel electrophoresis (SDS–PAGE) on a 12% polyacrylamide gel and transferred onto polyvinylidene fluoride (PVDF) membranes (Roche, Shanghai, China). The membranes were blocked with 5% nonfat milk in PBST, incubated with PEDV S-specific monoclonal antibodies (prepared in our lab), and subsequently incubated with anti-mouse IgG antibodies conjugated to HRP (Sigma-Aldrich Corporation, Saint Louis, MO, USA). Glyceraldehyde-3-phosphate dehydrogenase (GAPDH) protein served as the internal reference. Protein bands were detected using the ECL Chemiluminescence Detection Kit and Image Lab 4.0 software.

### 2.6. Animal Experiments

Weaned pigs and pregnant sows were purchased from the experimental farm of Huazhong Agricultural University. All experimental protocols were approved and conducted by the Laboratory Animal Ethical and Welfare Committee of Hubei Province, China (Approval No. 00314001).

#### 2.6.1. Safety Experiment

Twenty 21-day-old healthy piglets (negative for PEDV antigen and antibodies) were divided into four groups, with five piglets in each group. Group A received an intramuscular injection of 1 mL of 50 μg mRNA vaccine. Group B received an intramuscular injection of 1 mL of 75 μg mRNA vaccine. Group C received an intramuscular injection of 1 mL of 100 μg mRNA vaccine. Group D, the control group, was immunized with 1 mL of PBS. The safety of the mRNA vaccine was assessed by monitoring the temperature, appetite, mental state, injection site, and other adverse reactions in the immunized piglets. The total observation period is 21 days, with temperature recorded for 10 days.

#### 2.6.2. Analysis of Immunogenicity

PEDV-negative weaned pigs aged 3 to 4 weeks were randomly divided into four groups with five pigs per group. Group A received an intramuscular injection of 1 mL of 50 μg mRNA vaccine. Group B received an intramuscular injection of 1 mL of 75 μg mRNA vaccine. Group C received an intramuscular injection of 1 mL of 100 μg mRNA vaccine. Group D, the negative control group, was immunized with 1 mL of LNP-empty. A booster immunization with the same dose was administered 21 days postimmunization (DPI) for each group. For neutralizing antibody responses, blood samples were collected at 0, 21, 35, 60, and 90 DPI. Serum samples were tested for PEDV-specific neutralizing antibodies.

#### 2.6.3. Immune Challenge Test

The PEDV-negative pregnant sows were randomly assigned to two groups, each containing four sows. Group A received an intramuscular injection of 1 mL of 100 μg mRNA vaccine at −40 days and −20 days antepartum. In contrast, Group B, the negative control group, was given 1 mL of LNP-empty. Following immunization, blood samples were collected at 0, 21, and 35 days postimmunization to test for PEDV-specific neutralizing antibodies. Colostrum/milk samples were obtained from the sows at 0, 7, and 14 days to measure titers of PEDV-specific neutralizing antibodies. After production, piglets were randomly selected from immunized sows, fed infant formula milk every 4 h, and challenged with either the PEDV NMG20-3 strain (1 × 10^6.0^ TCID_50_/mL) or the NMG22-2 strain (1 × 10^6.0^ TCID_50_/mL) via the oral route. A challenged control group and the normal control group were also established.

### 2.7. Neutralization Test

The neutralization test was conducted as previously described [12,23]. Serum and milk samples were heat inactivated at 56 °C for 30 min. Subsequently, 50 microliters of serum samples were serially diluted twofold and mixed with 200 TCID50 of either PEDV NMG20-3 strain or NMG22-2 strain at 37 °C for 60 min. Serum–virus mixtures were added to confluent Vero cells cultured in 96-well plates, then the mixtures were removed after adsorption at 37 °C for 1 h. The plates were then washed for 10 min with PBS. Finally, 200 μL of serum-free DMEM medium containing trypsin (7.5 μg/mL) were placed into each well and maintained at 37 °C for 4 days. The cells were monitored for cytopathic effect (CPE). The neutralizing antibody titer was determined as the reciprocal of the highest serum dilution at which no CPE was observed. The reciprocal of the highest dilution that resulted in complete neutralization was defined as the neutralization titer.

### 2.8. Viral Shedding

The fecal swab samples were collected at 0, 1, 2, 3, 4, 5, 6, and 14 days post-challenge (DPC), and viral RNA was isolated using the TRIzol reagent (Invitrogen, Carlsbad, CA, USA) according to the manufacturer’s instructions. A real-time fluorescent quantitative PCR test was performed to detect PEDV as previously described [24]. The real-time PCR was conducted using the ABI ViiA7 system (Thermo Fisher Scientific, Waltham, MA, USA). Two primers (PEDV-F: 5′-ACGTCCCTTTACTTTCAATTCACA-3′ and PEDV-R: 5′-TACACACATCCAGAGTCA-3′) and one probe (FAM-5′-TGAGTTGATTACTGGCACGCCTA-3′-BHQ) were utilized for amplification. The real-time PCR reactions were carried out in a final volume of 25 μL, combining 5 μL of RNA template with a reaction mixture consisting of 12.5 μL of 2× multiplex RT-PCR buffer, 2.5 μL of 10× multiplex RT-PCR enzyme mix, and 1.5 μL of primer/probe mix (with a final concentration of 750 nM for each primer and 200 nM for the probe). Amplification occurred at 45 °C for 10 min, followed by reverse transcription at 95 °C for 10 min, and 40 cycles of 95 °C for 15 s and 60 °C for 45 s, during which fluorescence was collected. The number of viral RNA copies in fecal swabs was subsequently measured.

### 2.9. Histopathology and Immunohistochemistry

At 10 DPC, piglets from each group were euthanized. A complete necropsy of each animal was performed. Small intestine samples were collected and fixed in 10% neutral-buffered formalin. The duodenum, jejunum, and ileum were histopathologically examined using hematoxylin and eosin (HE) staining and immunohistochemistry (IHC) assays. In the IHC experiment, slides were blocked with 5% nonfat milk in PBS-T, incubated with PEDV S-specific monoclonal antibodies (prepared in our laboratory), and subsequently incubated with biotin-labeled anti-mouse IgG antibodies (Streptavidin–Biotin Complex Immunohistochemistry) (BOSTER, Wuhan, China). Positive signals were observed under an Olympus IX73 fluorescent microscope.

### 2.10. Statistical Analysis

Statistical analysis was conducted using GraphPad Prism (GraphPad Software 7.0, San Diego, CA, USA). All data were analyzed with one-way analysis of variance using GraphPad Prism software (Normality and Lognormality Tests). *p* < 0.05 was considered to be statistically significantly different.

## 3. Results

### 3.1. Construction of mRNA Vaccine

The PEDV S Protein of the NMG20-3 strain (G2c) was selected as the target antigen for the mRNA coding sequence. The truncated S gene was synthesized using codon optimization, resulting in the generation of two sequences. The pUC57-S01 and pUC57-S02 plasmids were linearized through BsPQI digestion and subsequently purified using a gel extraction kit. mRNA transcription was conducted using the purified linearized plasmid as a template with T7 RNA Polymerase. The quality of the mRNA was assessed by agarose gel electrophoresis. Two mRNA vaccines encoding PEDV S were prepared through encapsulation, specifically S01 and S02. The encapsulation efficiencies of mRNA-S01-LNP and mRNA-S02-LNP were approximately 88.5% and 87.6%, respectively. Particle size analysis indicated that the average particle sizes of mRNA S01 and S02 LNPs were 89.7 nm and 91.5 nm, respectively.

### 3.2. Expression of mRNA Vaccine

**Figure 2:**
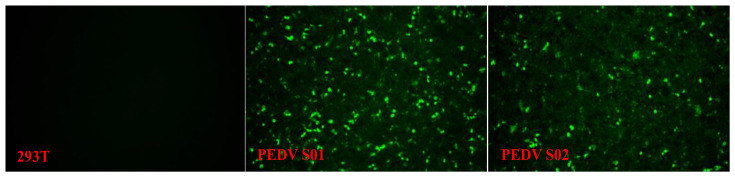
Indirect immunofluorescence analysis of expression of mRNA S01 and mRNA S02. HEK-293T cells were transfected with the mRNA S01 or mRNA S02, and lysate was analyzed by Indirect immunofluorescence. After 36 h post-transfection, the cells were fixed in 4% paraformaldehyde and were analyzed by IFA using the anti-PEDV S mAb as primary antibody and Alexa Fluor 488 goat anti-mouse IgG as secondary antibody. Fluorescence was observed under an Olympus IX73 fluorescent microscope.

To verify the expression of the mRNA vaccines (S01 and S02), HEK-293T cells were transfected with mRNA S01 and S02. Indirect immunofluorescence results showed that PEDV S-specific green fluorescence was observed in cells transfected with the mRNA vaccines S01 or S02 (Figure 2). No specific green fluorescence was detected in control cells. These findings indicated that the PEDV S protein was efficiently expressed in HEK-293T cells.

**Figure 3:**
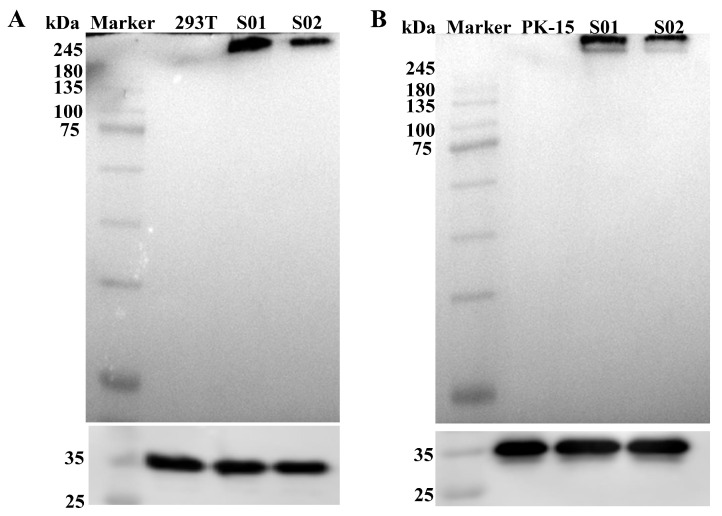
Western blot analysis of expression of mRNA S01 and mRNA S02. (**A**) HEK-293T cells were transfected with the mRNA S01 or mRNA S02, and lysate was analyzed by Western blotting. Lane 1: HEK-293T cells. Lane 2: mRNA S01. Lane 3: mRNA S02. (**B**) PK-15 cells were transfected with the mRNA S01 or mRNA S02, and lysate was analyzed by Western blotting. Lane 1: PK-15 cells. Lane 2: mRNA S01. Lane 3: mRNA S02.

To compare the expression of the S protein, HEK-293T and PK-15 cells were transfected with mRNA S01 and S02. Cell lysates were collected, and an immunoblot assay was performed using PEDV S-specific monoclonal antibodies. Specific protein expressions with the expected molecular weight (approximately 250 kDa) were detected in both mRNA-S01 and mRNA-S02 transfected cell samples (Figure 3). Notably, the expression of the S protein in mRNA-S01 was higher than that in mRNA-S02 when compared to GAPDH protein as an internal reference. These results suggest that the optimized sequence of mRNA S01 can significantly enhance antigen expression.

### 3.3. Safety of the mRNA S01

The safety of mRNA S01 was evaluated by monitoring temperature, appetite, mental state, injection site, and any adverse clinical reactions in immunized piglets across different doses. Temperature was measured for three days before and ten days after immunization.

**Figure 4:**
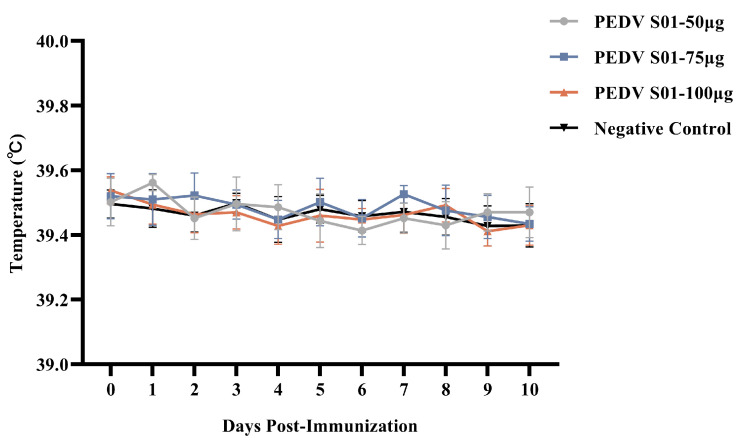
Characterization of the rectal temperature response induced by the mRNA S01 vaccine in piglets. The rectal temperature is measured by mercury thermometers.

The results indicated that the daily temperature of each immunized piglet remained below 40 °C (Figure 4), similar to that of the control group, within the normal range. During the 21-day observation period, all piglets survived and exhibited normal feeding behavior and mental states, with no adverse clinical reactions, good vaccine absorption, and no local abnormal reactions such as redness, swelling, or hard lumps at the injection sites, showing no significant differences compared to the control group. The safety of the mRNA S01 vaccine for piglets was demonstrated in animal safety testing.

### 3.4. Immunogenicity of the mRNA S01

**Figure 5:**
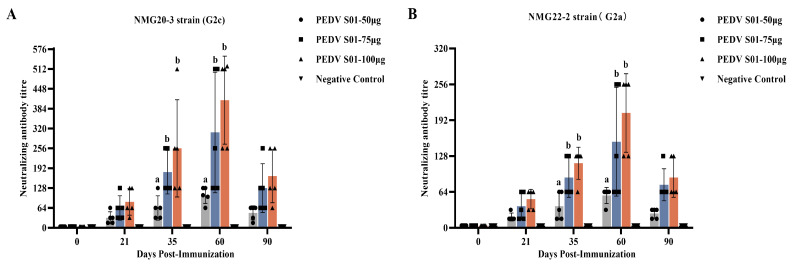
Detection of PEDV-specific neutralizing antibody in the sera of the immunized piglets. Neutralizing antibody titers against PEDV G2c NMG20-3 strain (**A**) and PEDV G2a NMG22-2 strain (**B**) were calculated. The neutralizing antibody titer was determined as the reciprocal of the highest serum dilution at which no CPE. The data were analyzed by using one-way ANOVA to compare the difference among groups immunized with different dose mRNA S01 at the same time. Different letters (a and b) indicate a statistically significant difference between different groups (*p* < 0.05).

The different doses of the mRNA S01 were evaluated in piglets, with LNP-empty serving as the control. Serum was collected at 0, 21, 35, 60, and 90 days postimmunization, and the levels of neutralizing antibodies were determined. All pigs vaccinated with mRNA S01 showed PEDV-specific neutralizing antibody responses at 21 days postimmunization, peaking at 60 days postimmunization. The neutralizing antibody titers induced by different immunization doses varied over time. At 35, 60, and 90 days postimmunization, the mean neutralization titers induced by mRNA S01 75 μg and mRNA S01 100 μg were higher than those induced by mRNA S01 50 μg. However, the differences in neutralizing titers between the mRNA S01 75 μg and mRNA S01 100 μg groups were not significant (Figure 5). The mean neutralization titers induced by mRNA S01 100 μg were higher than those of any other group. In contrast, no neutralizing antibody was detected in the sera of pigs in the control group. Furthermore, the titer of neutralizing antibodies was positively correlated with the increase in immunization dose. The heterologous cross-neutralizing antibody response was also evaluated using the PEDV (NMG22-2) strain of different genotypes. At 35 and 60 days postimmunization, the mean neutralization titers induced by mRNA S01 were higher against the homologous virus strain than against the heterologous virus strain. The antibody levels of all vaccinated pigs peaked at 60 d, with mean neutralizing antibody titers of 1:58, 1:154, and 1:205, respectively. At 35, 60, and 90 dpi, the antibody titers induced in the piglets vaccinated with mRNA S01 75 μg and mRNA S01 100 μg were significantly higher than those induced in the piglets vaccinated with mRNA S01 50 μg (*p* < 0.05). However, the difference between mRNA S01 75 μg group and mRNA S01 100 μg group was not significant (*p* > 0.05). These results confirm that piglets immunized with mRNA S01 75 μg and mRNA S01 100 μg elicited high-level immune responses and showed enhanced cross-reactive neutralizing antibodies against both homologous and heterologous virus strains.

### 3.5. Protective Efficacy of the mRNA S01 Against PEDV

**Figure 6:**
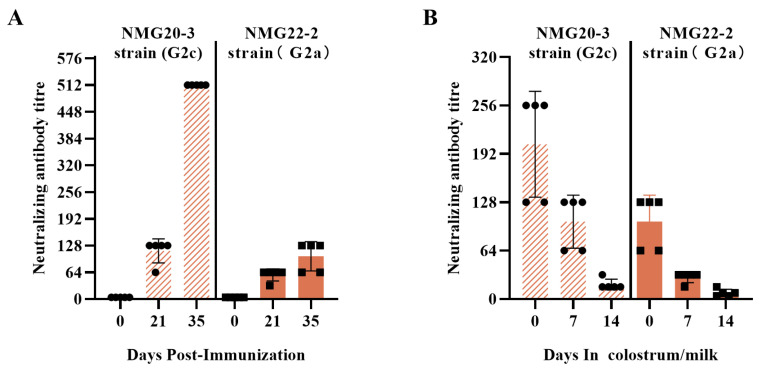
Detection of PEDV-specific neutralizing antibodies in the sera and colostrum/milk of sows. (**A**) Serum neutralizing antibody titers against PEDV. (**B**) Colostrum/milk neutralizing antibody titers against PEDV. The neutralizing antibody titer was determined as the reciprocal of the highest serum dilution at which no CPE.

To further evaluate the immunogenicity of the mRNA S01 in pigs, pregnant sows were immunized with 100 μg of mRNA S01 at -40 days and -20 days antepartum. The mRNA S01 vaccine was safe, with no observed deaths or other adverse effects at the injection site during the observation period. As shown in Figure 6, mRNA S01 induced high titers of NAbs in serum and colostrum/milk samples. At 35 DPI, the mean neutralization titers were 1:512 against the homologous NMG20-3 strain (Figure 6). Conversely, the mean neutralization titers were 1:102 against the homologous NMG22-2 strain.

Similarly to the trend obtained from experiments in piglets, the highest levels of neutralizing antibodies were generated against the homologous NMG20-3 strain, whereas the neutralization ability of antibodies against the heterogeneous NMG22-2 strain was lower. These results indicate that the titers of neutralizing antibodies and cross-neutralizing antibodies in breast milk gradually decrease.

To assess the protective efficacy of the mRNA S01 vaccine against PEDV, five-day-old piglets from the mRNA S01 immunization group and the control group were challenged with PEDV NMG20-3 strain or NMG22-2 strain via the oral route. Clinical signs, histological lesions, and viral shedding were evaluated. In the LNP-empty group, all piglets displayed typical clinical signs such as watery diarrhea and weight loss, and all piglets died at 7–10 DPC. After being challenged with the PEDV NMG20-3 strain, piglets in the mRNA S01 group did not show any clinical symptoms. After challenge with the PEDV NMG22-2 strain, two piglets in the mRNA S01 group exhibited mild diarrhea at 5–6 DPC.

**Figure 7:**
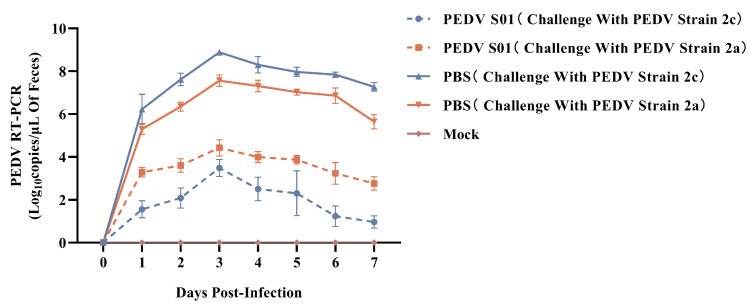
Viral shedding of the piglets after PEDV challenge. After PEDV challenge, viral shedding was detected by real-time PCR. All data are expressed as mean ± SEM.

Fecal swab samples were collected after the challenge, and viral shedding was detected by RT-qPCR. In the LNP-empty group, PEDV RNA was detected in all fecal swabs, peaking at 3 DPI. The PEDV shedding in the fecal swabs of the mRNA S01 group was significantly lower than that of the LNP-empty group. As shown in Figure 7, after challenge with the PEDV NMG22-2 strain (G2a), the virus copy numbers in the mRNA S01 group were higher than those in the vaccinated group challenged with the PEDV NMG20-3 strain (G2c).

Autopsies were performed on all deceased and surviving piglets. No apparent gross lesions were observed in the mRNA S01 group challenged with the NMG22-2 strain. However, the intestinal tubes of two of the five piglets in the mRNA S01 group were dilated and contained a significant amount of yellow serous fluid. All piglets in the LNP-empty group exhibited dilated intestinal tubes, which had thinner, transparent walls and also contained a large amount of yellow serous fluid. Histopathological and immunohistochemical analyses were subsequently conducted.

**Figure 8:**
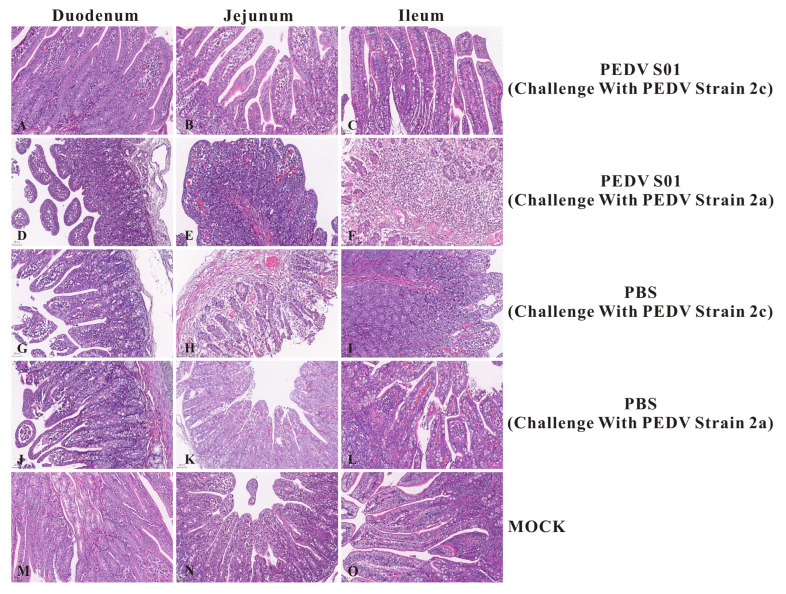
Histopathological examination of the duodenum, jejunum, and ileum of piglets after PEDV challenge. Severe pathological changes, including significant atrophy and shedding of the duodenum, jejunum, and ileum of piglets in the control group (PBS). No significant histological lesions were observed in the mRNA S01 group. Original magnification is ×200.

**Figure 9:**
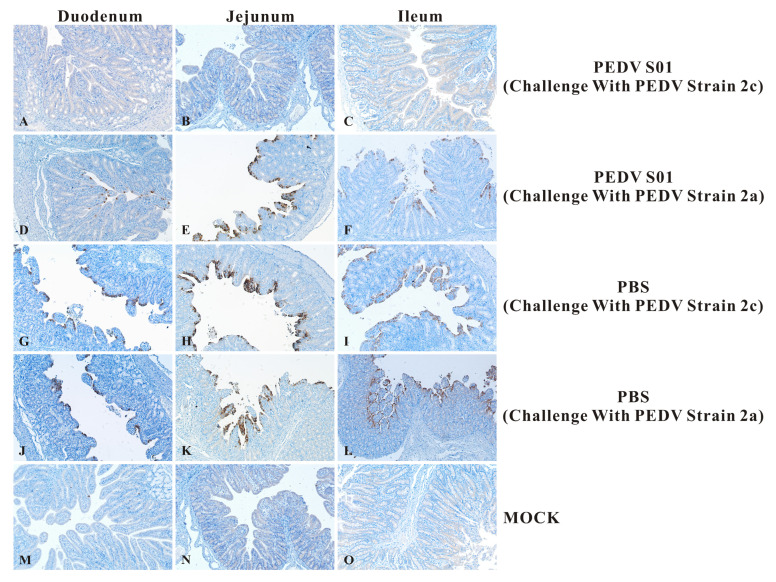
Immunohistochemical analysis of the duodenum, jejunum, and ileum of piglets after PEDV challenge. The IHC results revealed significant positive PEDV antigen signals in the intestinal epithelial cells of the duodenum, jejunum, and ileum in the control group (PBS). No positive PEDV antigen signals were detected in the mRNA S01 group challenged with the PEDV NMG20-3 strain.

Histopathological analysis indicated that all piglets in the LNP-empty group experienced severe pathological changes, including significant atrophy and shedding of the duodenum, jejunum, and ileum. The intestinal villi epithelial cells were swollen and necrotic, accompanied by minimal lymphocyte infiltration (Figure 8). In contrast, no pathological changes were identified in the intestines of the mRNA S01 group challenged with the PEDV NMG20-3 strain and the normal control group (MOCK). The IHC results revealed significant positive PEDV antigen signals in the intestinal epithelial cells of the duodenum, jejunum, and ileum in the LNP-empty group (Figure 9). No positive PEDV antigen signals were detected in the mRNA S01 group challenged with the PEDV NMG20-3 strain and the normal control group (MOCK). Collectively, these results demonstrate that the mRNA S01 vaccine provided newborn piglets with effective immunity, protecting them against the homologous PEDV strain challenge. In contrast, the mRNA S01 vaccine conferred only partial protection against heterologous virus strains.

## 4. Discussion

mRNA vaccine technology, as an innovative approach to vaccine development, shows considerable potential in preventing viral infectious diseases. Compared with traditional vaccines, such as attenuated live vaccines and DNA vaccines, mRNA vaccines primarily consist of in vitro transcribed (IVT) mRNA, eliminating common endotoxins and other infection risks associated with conventional vaccines. The rapid development and deployment of COVID-19 mRNA vaccines have offered valuable insights for epidemic control [25]. Unlike traditional vaccine development, mRNA vaccines can be designed and produced in a very short timeframe, providing significant advantages for managing emerging infectious diseases. The creation of safe and efficient delivery systems remains essential for mRNA vaccine research. LNPs have gained widespread acceptance for mRNA vaccine delivery due to their safety and high efficiency. LNPs typically consist of four components: ionizable cationic lipids, PEGylated lipids, cholesterol, and helper phospholipids [17,19].

PED is the most severe threat to nursing piglets (under 2 weeks old), resulting in a 100% morbidity rate. As an RNA virus, PEDV has a high probability of molecular evolution and mutation [7,26,27]. The S gene, responsible for encoding the highly mutable S protein, serves as an indicator gene for viral virulence. Variations and recombinations in the S gene may alter PEDV pathogenicity and generate novel strains. Currently, PEDV G2a and G2c are the main epidemic strains in China [7]. The PED control strategy primarily relies on immunization, where domestically developed combined live vaccines or inactivated vaccines against PEDV and TGEV have played significant roles. However, the prevalent strains of PEDV recently isolated from pigs in China have been shown to mutate rapidly, and existing products have failed to provide adequate protection. Therefore, developing new PED vaccines or multipartial vaccines based on the prevalent strains of PEDV has become an urgent demand for the pig industry in China. There is a lack of systematic research on the cross-identification and protection differences among different PEDV genotypes. Understanding these differences is crucial for developing more effective vaccines.

The full-length S protein contains multiple antigenic epitopes, which can simultaneously induce neutralizing antibodies and T-cell immune responses targeting different regions, thereby enhancing the broad spectrum and durability of immune protection. The trimeric structure of the S protein presents specific conformations (such as the pre-fusion conformation) during natural infection [11,28]. Using the full-length S gene can preserve its natural conformation, making the antibodies produced by the vaccine more similar to the immune response during actual viral infection. Therefore, the full-length S protein antigen exhibits higher immunogenicity. This strategy has been validated by the success of COVID-19 vaccines.

Vaccination is a key strategy for preventing and controlling PEDV outbreaks. However, the high variability of the S gene often compromises vaccine efficacy. Clinical cases have already confirmed the lack of effective immunological cross-protection among different PEDV genotypes. In China, the existing commercial vaccines of the main strain is for the PEDV 2b strain, which cannot effectively protect against the prevalent strains (PEDV G2a strain or PEDV G2c strain). Yongxiang Zhao et al. (2024) reported that the PEDV-S mRNA-LNP vaccine is a promising candidate for combating PEDV infection, in which the design concept of mRNA vaccine is different from ours [29]. Furthermore, the AH2012/12 strain of PEDV (GenBank: KC210145) is no longer the prevalent strain in China. This study is the first to report a novel mRNA vaccine of PEDV G2c strain.

In this study, mRNA encoding the S proteins of PEDV was successfully expressed in vitro. Notably, the expression of the S protein in mRNA-S01 was found to be higher than that in mRNA-S02. Ultimately, the safety and immunogenicity of mRNA-S01 were evaluated through animal experiments. The results indicated the safety of the mRNA-S01 vaccine for piglets and pregnant sows. This study demonstrated that the mRNA-S01 vaccine effectively induces humoral immunity in a dose-dependent manner. Piglets and pregnant sows immunized with the mRNA-S01 vaccine developed significantly higher PEDV-specific neutralizing antibody titers to homologous strains compared to heterologous strains. These results suggest antigenic variations between genotype G2c and genotype G2a of the PEDV S protein. Challenge protection efficiency is a critical criterion for evaluating vaccine efficacy. The results of challenge protection efficiency showed that the mRNA-S01 vaccine conferred effective immunity to newborn piglets, protecting them against the homologous PEDV strain challenge.

## 5. Conclusions

In conclusion, we successfully prepared a PEDV mRNA-S01 vaccine that induces long-lasting neutralizing antibodies and provides effective protection against the homologous PEDV strain challenge. Our results present a novel approach for the further development of PEDV mRNA vaccines.

## Data Availability

The datasets used and analyzed during the current study are available from the corresponding author on reasonable request.

## Supplementary Materials

The following supporting information can be downloaded at: https://www.mdpi.com/article/10.3390/v17091210/s1, Figure S1. The isolation and identification of PEDV the NMG20-3 and NMG22-2 strain. (A) Phylogenetic analysis based on the S gene of the PEDV strain. (B) CPE of PEDV the NMG20-3 and NMG22-2 strain in Vero cells. (C) IFA of PEDV the NMG20-3 and NMG22-2 strain in Vero cells.
