# Supplementary material for: Development of a Safe and Effective mRNA Candidate Vaccine Against PEDV G2c Genotype Infection
Source: Viruses. 2025 Sep 4;17(9):1210. doi: 10.3390/v17091210 (PMC12474126; doi:10.3390/v17091210)
Supplement: Supplementary file 1 [file viruses-17-01210-s001.zip › viruses-3836296-supplementary.pdf]

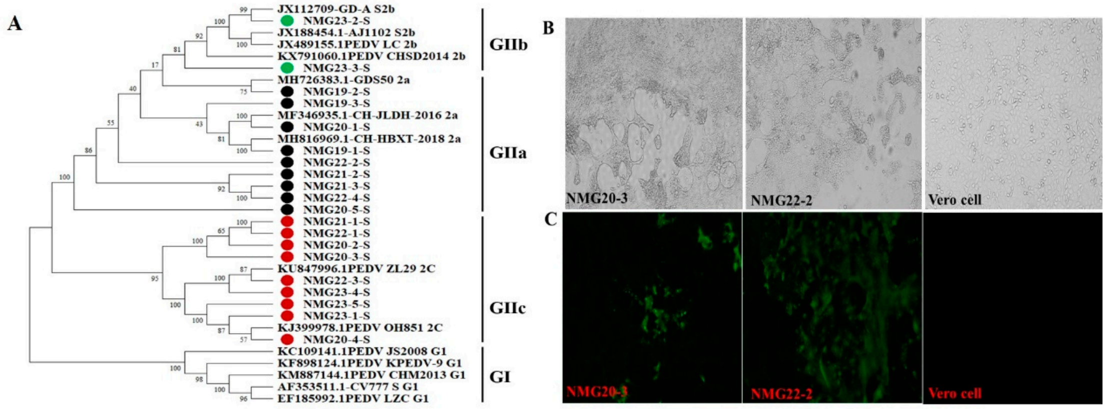

Figure S1. The isolation and identification of PEDV the NMG20-3 and NMG22-2 strain. (**A**) Phylogenetic analysis based on the S gene of the PEDV strain. (**B**) CPE of PEDV the NMG20-3 and NMG22-2 strain in Vero cells. (**C**) IFA of PEDV the NMG20-3 and NMG22-2 strain in Vero cells.
